# Resuscitative Endovascular Balloon Occlusion of the Aorta in trauma: a systematic review of the literature

**DOI:** 10.1186/s13017-017-0153-2

**Published:** 2017-08-29

**Authors:** Emiliano Gamberini, Federico Coccolini, Beatrice Tamagnini, Costanza Martino, Vittorio Albarello, Marco Benni, Marcello Bisulli, Nicola Fabbri, Tal Martin Hörer, Luca Ansaloni, Carlo Coniglio, Marco Barozzi, Vanni Agnoletti

**Affiliations:** 1 Anesthesia and Intensive Care Department, AUSL Romagna Trauma Center “Maurizio Bufalini” Hospital, Viale Ghirotti 286, 47521 Cesena, Italy; 2 General and Emergency Surgery Department, ASST Trauma Center “Papa Giovanni XXIII” Hospital, Piazza OMS 1, 24127 Bergamo, Italy; 3 Emergency Medicine, University of Modena and Reggio Emilia, via Università 4, 41121 Modena, Italy; 4 Interventional Radiology Department, AUSL Romagna Trauma Center “Maurizio Bufalini” Hospital, Viale Ghirotti 286, 47521 Cesena, Italy; 5 General and Emergency Surgery Department, AUSL Romagna Trauma Center “Maurizio Bufalini” Hospital, Viale Ghirotti 286, 47521 Cesena, Italy; 6 Cardiothoracic and Vascular Surgery Department, Örebro University Hospital, Södra Grev Rosengatan, 701 85 Örebro, Sweden; 7 Anesthesia, Intensive Care and 118 Emergency System Department, AUSL Bologna Trauma Center “Maggiore” Hospital, Largo Nigrisoli 2, 40133 Bologna, Italy; 8 Emergency Medicine Department, AUSL Modena Trauma Center “Sant’Agostino” Hospital, Via Pietro Giardini 1355, 41126 Modena, Italy

**Keywords:** REBOA, Aortic balloon occlusion, Hemorrhagic shock, Severe trauma, Trauma system, Trauma center, Bleeding, Systematic review

## Abstract

**Aims:**

Resuscitative endovascular balloon occlusion of the aorta has been a hot topic in trauma resuscitation during these last years. The aims of this systematic review are to analyze when, how, and where this technique is performed and to evaluate preliminary results.

**Methods:**

The literature search was performed on online databases in December 2016, without time limits. Studies citing endovascular balloon occlusion of the aorta in trauma were retrieved for evaluation.

**Results:**

Sixty-one articles met the inclusion criteria and were selected for the systematic review. Overall, they included 1355 treated with aortic endovascular balloon occlusion, and 883 (65%) patients died after the procedure. In most of the included cases, a shock state seemed to be present before the procedure. Time of death and inflation site was not described in the majority of included studies. Procedure-related and shock-related complications are described. Introducer sheath size and comorbidity seems to play the role of risk factors.

**Conclusions:**

Resuscitative endovascular balloon occlusion of the aorta is increasingly used in trauma victim resuscitation all over the world, to elevate blood pressure and limit fluid infusion, while other procedures aimed to stop the bleeding are performed. High mortality rate is probably due to the severity of the injuries. Time and place of balloon insertion, zone of balloon inflation, and inflation cutoff time are very heterogeneous.

## Background

Hemorrhagic shock is a major cause of death [1, 2]. Although the main aims of resuscitation are to stop the hemorrhage and restore circulating blood volume, persistent hemorrhage can be rapidly fatal. In major trauma, uncontrolled bleeding is the first cause of potentially preventable death [3–5]. Resuscitative endovascular balloon occlusion of the aorta (REBOA) has been used in a variety of clinical settings (postpartum hemorrhage, upper gastrointestinal hemorrhage, pelvic hemorrhage during pelvic/sacral tumor surgery, traumatic abdomino-pelvic hemorrhage, ruptured aneurysm abdominal aorta [6–9]) to successfully elevate central blood pressure in the setting of shock, even if the evidence base is weak and devoid of clear indications. The effectiveness in this clinical target seems to have been confirmed by recent pooled analyses [10] that demonstrated an increase in mean systolic pressure following REBOA use; however, benefits in terms of overall reduction of trauma patient mortality are controversial [11, 12] (Table 1). Prospective data collection is underway in the form of an American Association for the Surgery of Trauma-sponsored observation study [13] and a European registry [14] which should permit the consistent recording of REBOA-specific data, including indications and outcome. The aortic level of balloon inflation is usually reported according to the three zone classifications: zone I thoracic aorta from left subclavian and celiac artery, zone II between celiac and renal artery, and zone III infra-renal placement [15, 16]. For bleeding in the abdominal cavity, the REBOA balloon is placed in zone I. For pelvic bleeding, generally from iliac artery branches, the balloon is placed in the distal aorta (zone III). Zone II is not currently in use. Prophylactic balloon placement in hemodynamically stable patients at risk of significant hemorrhage [10] has also been described. Positioning could lead to device-related morbidity (3.7%) and mortality (0.8%) due to arterial perforation or dissection, insertion site bleeding, and balloon-related thromboembolic events [10].

**Table 1:** Report of included papers regarding REBOA in trauma, in chronological order, except for the last three to whom full text was recovered from other sources

Ref.	Year	Authors	Study type	No. of patients	Zone	Shock	Mortality
[26]	2016	Okada Y	Case report	1	I	Y	0/1 0%
[30]	2016	Uchino H	Case report	1	–	Y	1/1 100%
[28]	2016	Sadek S	Case report	1	III	Y	0/1 0%
[34]	2016	Matsumoto N	Case report	1	I	Y	0/1 0%
[29]	2016	DuBose JJ	Prospective observational study	46	8 × III 1 × II 33 × I 4 × converted to aortic open occlusion	Y	33/46 72%
[20]	2016	Costantini TW	Prospecting observational multicenter study	5	–	Y	3/5 60%
[5]	2016	Tsurukiri J	Retrospective study	13	4 × III 3 × II 6 × I	Y	3/13 23% Emergency room 6/13 46.1% 24 h 7/13 53.8% 60 g
[17]	2016	Inoue J	Retrospective cohort study	625	–	Y	386/625 61.8% intrahospital
[35]	2016	Hörer TM	Case series	3	1 × II 2 × I	Y	1/3 33.3%
[21]	2016	Hörer TM	Case series	7	–	Y	0/7 0%
[12]	2015	Moore LJ	Retrospective cohort study	24	5 × III 19 × I	Y	15/24 62.5%
[18]	2015	Saito N	Retrospective cohort study	24	I	Y	10/24 41.7% 24 h 17/24 70.8% 30 days
[11]	2015	Norii T	Observational prospective study	452	–	Y	343/452 75.9%
[19]	2015	Irahara T	Retrospective observational study	14	–	Y	9/14 64.3%
				1		N	0/1 0% TOT: 9/15 60%
[27]	2015	Ogura T	Case series	7 angioembolization + REBOA	–	Y	1/7 14.2% 28 days
				35 REBOA + other treatments			16/35 46%
[36]	2013	Brenner ML	Case series	6	3 × III 3 × I	Y	2/6 33.3%
[31]	2010	Martinelli T	Case series	13	III	Y	7/13 53.8%
[37]	2009	Kataoka Y	Case series	3	–	Y	2/3 66.7%
[38]	2004	Long JA	Case report	1	–	Y	–
[39]	2003	Linsenmaier U	Case series	3	III	Y	2/3 66.7%
[40]	1995	Segol P	Case series	3	I	–	1/3 33.3%
[41]	1989	Gupta BK	Case series	21	I	Y	14/21 66.7%
[42]	1986	Low RB	Case series	15	–	Y	13/15 87%
[43]	1986	Wolf RK	Case report	1	III	Y	0/1 0%
[44]	1954	Hughes CW	Case series	2	I	Y	2/2 100%
[22]	2001	Matsuoka S	Case report	1	I	Y	0/1 0%
[32]	2016	Teeter WA	Retrospective review	33	I	Y	17/33 51% 24 h 19/33 58% 30 days

This aims to provide a systematic analysis of currently available literature regarding the use of REBOA in trauma victims.

## Materials and methods

The methodological approach includes the development of selection criteria, definition of search strategies, and abstraction of relevant data. The PRISMA statement checklist for reporting a systematic review was followed.

### Types of study included and criteria selection

All studies concerning REBOA use in trauma were retrieved and analyzed.

Review articles, systematic reviews, interventional trials, case series, and reports were considered eligible for inclusion in this systematic review. Conference abstracts, letters, experimental papers with animals, and commentaries were not considered.

### Types of participants and intervention

Trauma victims who underwent REBOA during emergency department (ED) and operating room (OR) resuscitation phase were considered.

### Types of outcome measures

The primary outcome was hospital mortality. All secondary parameters reported in the selected studies were evaluated.

### Literature search and selection

Literature search was performed online on MEDLINE (through PubMed) and Cochrane Oral Health Group Specialized Register, with the addition of five articles identified from references of other works.

In order to facilitate the identification of relevant articles, the research equation was based on the following text words and criteria: “REBOA” or “resuscitative endovascular balloon occlusion of the aorta” or “ABO” or “aortic balloon” and “trauma” as *title/abstract*.

The literature search was performed in December 2016, with no time limit.

**Fig. 1:**
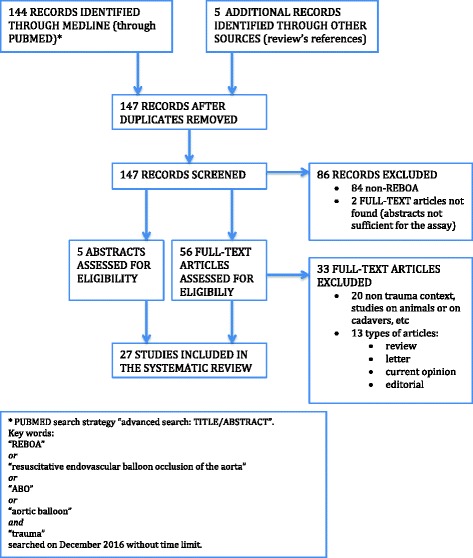
Flow chart of the study search, selection, and inclusion/exclusion

Out of the 144 initially identified articles, 61 met the inclusion criteria and were selected for the systematic review, 28 of which were actually analyzed for outcome measures and included in this review. The flow chart of study identification and the inclusion/exclusion process is shown in Fig. 1 *.*

It was not possible to recover the full text of some articles; however, these have been included in the table (but not commented), when the data necessary for our analysis could be extrapolated from the abstracts.

### Study characteristics/results

One thousand three hundred fifty-five patients treated with REBOA were included in this systematic review. Most of them were in a state of shock when REBOA was positioned; mortality was 65% (883 patients) and time of death was not always reported.

One hundred forty-nine patients were treated with REBOA in zone I, 5 in zone II, and 38 in zone III. In the majority of cases, REBOA zone was not described.

### Criteria of inclusion/exclusion of patients

The studies were carried out mostly on an adult population (namely patients aged over 15 [5] or 16 [12, 17] or 18 years old, according to the explicit indications).

The studies are retrospective and used registries, so they refer to the time period in which the cases were selected (e.g., 2014 or from January 2007 to December 2013).

Inclusion criteria are heterogeneous. Patients included in the studies were analyzed for uncompressible trunk hemorrhage, intra-abdominal bleeding (e.g., liver or splenic injury), retroperitoneal hemorrhage (e.g., renal injury or pelvic fracture), and non-traumatic hemorrhage (e.g., obstetric or gastrointestinal that have been excluded from this review).

Patients’ selection criteria vary depending on the studies, and they are usually indicated for example hemoperitoneum or pelvic ring fractures with potential imminent cardiac arrest or fluid resuscitation unresponsiveness state with a sustained SBP of less than 90 mmHg [18]. Some studies excluded patients who went into cardiac arrest during admission, were diagnosed with any terminal disease during the study period [5], or sustained what were defined as un-survivable injuries [17].

Lastly, some research excluded patients where REBOA was positioned to prevent shock (still hemodynamically stable); in the queue of their article, Irahara et al. [19] described an interesting *case report* regarding the prophylactic use of REBOA in potentially evolutionary cases. While this practice is of great interest, other authors have confessed fears that they may become too invasive [18].

Many studies include patients undergoing treatment with REBOA for reasons other than trauma (e.g., bleeding of the gastrointestinal tract, post-partum bleeding); those only dealing with REBOA in non-traumatic cases were eliminated in the initial selection of this systematic review (Fig. 1), whereas articles including trauma patients were analyzed to extract data of interest.

### Definition of shock

Some studies provided an explicit definition of shock (e.g., SBP < 90 mmHg or SI ≥ 1 [5]; SBP < 90 mmHg or HR > 120 bpm or base deficit < − 5 [20]; SBP < 80 mmHg and no response to fluid treatment [21]) while others did not; however, it was possible to deduce patient’s vital parameters from the tables (mean patient SBP was often indicated in the tables, and for the purposes of this review, < 90 mmHg was defined as shock).

### Vascular approach

Almost all REBOA was introduced from the femoral artery (which is considered as the ideal access point). Other access points are described in a limited number of cases described (e.g., left brachial artery or left common carotid artery [22]).

Access can be achieved using different techniques: percutaneous (with the Seldinger method), open exposure by surgical cutdown of the vessel, or exchange over a guidewire from an existing arterial line [16].

Imaging techniques can be used (e.g., eco guide for access and RX to confirm the balloon position), or it is also possible to work blindly, using only external landmarks; MacTaggart et al. describe the use of “morphometric roadmaps” to improve accurate device delivery for fluoroscopy-free REBOA, according to the patient physique [23].

REBOA insertion time in the simulation laboratory was under 5 min (Brenner et al.) [12–24]. Simulations were often videotaped in the *trauma center*, with a few performances under 3 min and some lasting up to 15 min when femoral access was difficult to obtain [12].

### Operator and training

Operators who insert REBOA vary depending on the context; in a multidisciplinary team, the practice may be performed by an interventional radiologist, a vascular surgeon, a trauma surgeon, an intensive care unit expert, or an emergency department physician who is familiar with the endovascular approach.

The skill can be quickly acquired; for physicians with limited previous endovascular skills, attendance at procedural courses provides an opportunity to gain competence and it seems that the insertion of no. 3–5 REBOA under expert supervision [5], or a training period of some months, can be enough [11, 12].

There is no universal certification for REBOA positioning. For example, in order to become an emergency physician, the Japanese Association for Acute Medicine requires a minimum experience of no. 3 cases of REBOA insertion during residency training. It does not differ from the US “BEST” (Basic Endovascular Skills for Trauma) courses which include didactic lectures, virtual reality simulation, and cadaveric instruction, whereas “ESTARS” (Endovascular Skills for Trauma and Resuscitative Surgery) courses use simulations and live animal models to establish procedural competence [25].

There are also some courses in Europe, especially in London (Royal Medical Hospital and London’s Air Ambulance) and in Örebro, Sweden (“EVTM” EndoVascular and hybrid Trauma and bleeding Management).

### Type of device

In most of the studies, a 10 to 12 Fr introducer is used (Okada et al. considered the large size as a contributory cause of ischemic complications that led to lower limb amputation patients with a smaller build [26]).

Seven-French catheters are currently available (since 2014) and in use in Japan; Tsurukiri et al. have noted that this has enabled a reduction in complications coupled with 100% technical success [5].

### Time and method of inflation

An ideal time of occlusion has not been established, although it is clear that it must be as short as it possible. There are several studies on animals (especially pigs and dogs) which identify 60–90 min as a cutoff time; however, it is difficult to adapt these data for human patients. Saito et al. define the golden time as 20 min of hemostasis as a goal for the future [18].

Some of the analyzed retrospective studies do not report occlusion time as it had not been recorded. However, it was found that occlusion time was shorter in survivors than in patients who died [19]. Mean occlusion time in the various articles ranged from 20 to 65 min.

During the CT scan, the balloon is partially deflated to let the contrast pass. It is interesting to note that when occlusion must necessarily exceed 20 min, Ogura et al. practiced a partial deflation for a few minutes and completed a rapid transfusion of blood products in order to take advantage of the ischemic preconditioning effect as a strategy to increase tissue tolerance. During this interval, if SBP was not maintained above 70 mmHg, the balloon was inflated again for a further 20 min. In this study, the total mean occlusion time is 80 min (although one patient did not tolerate deflation) and there were no complications related to the use of REBOA [27]. However, this approach does not seem to bring other advantages.

A significant correlation between total occlusion time, serum lactate concentration, and the shock index was noted [5].

The sheath can be left in place until it has been established with certainty that it is no longer required (although this could cause ischemic complications, especially patients with a smaller build [26]).

The development of new devices that do not require an oversized sheath or long guidewires is likely to reduce not only complications but also time to occlusion [5].

### Insertion setting

REBOA is mostly positioned in emergency departments, in some cases in the operating room or, where available, in a hybrid room. There is also REBOA experience in pre-hospital settings (e.g., London’s Air Ambulance, although limited to blind positioning in zone III only [28]).

### Mortality (when, where, why)

As for mortality, several aspects must be considered: when, where (emergency department, operating room, intensive care unit), and why.

Some studies report mortality percentages with precise temporal references (e.g., mortality at 24 h, 30 days, 60 days, etc.), while others do not.

Saito et al. claim that mortality within 24 h is more relatable to REBOA because after this time, patients die from other causes [18].

Many studies compare the mortality of patients treated with REBOA versus resuscitative thoracotomy, matching patients with similar characteristics; most of them do not report favorable data for REBOA. However, Moore et al. [12] noted that the deaths of *REBOA patients* appear to be delayed and typically occur when the patient is already out of the emergency department and due to complications other than bleeding (especially multi-organ failure and brain injury). Post-REBOA deaths more commonly occurred in emergency or operatory room while a significantly larger portion of post-open aortic occlusion occurred in the intensive care unit [29].

### Complications

In several series of patients, no complications have been related to the use of REBOA.

Eventually, complications may be related to the insertion, to the REBOA mechanism (pressure increases upstream from the occlusion), or to the failure of the technique itself.

Described complications are:

- 0.66% distal ischemia/thromboembolic events [18, 21, 26, 29] (with eventual need for amputation) [26]
- 0.07% intracranial massive hemorrhage [30]
- 0.22% pseudo-aneurysm in the access site [29]/arterial injury caused by puncture [18]
- 0.89% kidney failure [18]
- Spinal cord ischemia (no REBOA cases) [18]
- 0.15% balloon migration (e.g., in zone II) [29]
- 0.30% infections [29]
- 0.07% retroperitoneal hematoma following the blind insertion (the vessel was repaired a few days later without further complications); this is more common in obese patients in whom multiple attempts are performed; ultrasound-guided procedure, thanks to the widespread of portable devices, is useful as long as this does not cause an excessive elongation of the time [18, 21]
- 0.66% introducer insertion failure: especially in elderly patients (over 75 years, above all females) who had subsequently undergone resuscitative thoracotomy or REBOA in the angiography room and where the angiography revealed severe tortuosity or twisting of the femoral artery. [5]
- 0.07% rupture of the balloon (immediately replaced) [31]

The main risk factors are high body mass index, thrombocytopenia, emergency procedures, big size of the introducer, and use of anti-platelet drugs [5]. Complications seem to reduce significantly with 7-Fr catheter [32].

### REBOA in combination with other techniques

REBOA is not considered as a permanent solution, rather it constitutes a bridge for patient stabilization until definitive hemostasis (angioembolization, surgery, or hybrid technique), eventually achieved during the diagnostic completion techniques (TC) and transfers.

However, in some circumstances, it was not enough by itself and it has been used in conjunction with other techniques to control bleeding, e.g., external fixator and pelvic packing. Sometimes, it was necessary to convert to an aortic open occlusion (e.g., 4 of 46 patients described by DuBose et al. [29]); other times, REBOA has been used in patients who have already undergone resuscitative thoracotomy; however, such cases have been excluded from the study (Saito et al. [18]) and from this review.

Table 1 shows the list of papers included, in chronological order, except for the last three to whom full text was recovered from other sources.

## Conclusions

Resuscitative endovascular balloon occlusion of the aorta has been used increasingly during the last 10 years to elevate central arterial blood pressure in severely injured trauma victims with abdominal and/or pelvic bleeding, limiting infused fluid volume. Studies included in this review showed huge heterogeneity in patient selection, procedure performing time and environment, and balloon deflation cutoff time. However, there is homogeneity in using REBOA in severely injured patients using femoral artery access, with the aim to transiently stop or reduce distal aortic blood flow, while various procedures to finally control the bleeding are performed. Direct REBOA-related complications seem to have a minor role on mortality and are limited to local vascular injuries. High mortality rate is a feature of severely multiple injured patients, and REBOA role as a bridge to final bleeding control clearly emerges, even though not always effective. Pre-hospital REBOA role in trauma victim resuscitation, partial or intermittent balloon inflation, “prophylactic” REBOA insertion in selected cases, REBOA in combination with resuscitative thoracotomy for witnessed traumatic cardiac arrest, and resuscitative endovascular balloon occlusion of inferior vena cava to treat injuries in this site, should be interesting issues for the next future. Taking into account worldwide huge heterogeneity in trauma team composition and setting, considering also available literature concerning REBOA flow charts [33], an updated Trauma System should equip oneself of specific REBOA algorithm, included in severe trauma resuscitation protocol, accordingly with his own features. Different Trauma System benchmark will be the way to better understand and perform in severe hemorrhagic trauma resuscitation.

## Abbreviations

ED: Emergency department
REBOA: Resuscitative endovascular balloon occlusion of the aorta
